# Role of lateral suspension for the treatment of pelvic organ prolapse: a Delphi survey of expert panel

**DOI:** 10.1007/s00464-024-10917-5

**Published:** 2024-06-14

**Authors:** Tommaso Simoncini, Andrea Panattoni, Tina Cadenbach-Blome, Nicola Caiazzo, Maribel Calero García, Marta Caretto, Fu Chun, Eric Francescangeli, Giorgia Gaia, Andrea Giannini, Lucas Hegenscheid, Stefano Luisi, Paolo Mannella, Liliana Mereu, Maria Magdalena Montt-Guevara, Isabel Ñiguez, Ratiba Ritter, Eleonora Russo, Maria Luisa Sanchez Ferrer, Ayman Tammaa, Bernhard Uhl, Bea Wiedemann, Maciej Wilczak, Friedrich Pauli, Jean Dubuisson

**Affiliations:** 1 Division of Obstetrics and Gynecology, Department of Clinical and Reproductive Medicine, University of Pisa, via Roma 67, 56126 Pisa, Italy; 2 AOU Pisana: Azienda Ospedaliero Universitaria Pisana, Pisa, Italy; 3 Asklepios Klinik Altona, Hamburg, Germany; 4 Division of Obstetrics and Gynecology, Ospedale Giovanni Battista Grassi di Ostia, Azienda ASL Roma 3, Rome, Italy; 5 Division of Obstetrics and Gynecology, Hospital Materno-Infantil Quirónsalud, Seville, Spain; 6 Department of Obstetrics and Gynecology, The Second Xiangya Hospital Central South University, Changsha, Hunan China; 7 Division of Obstetrics and Gynecology, Istituto Clinico Sant’Anna, Brescia, Italy; 8 Department of Gynecology, ASST Santi Paolo e Carlo, Milan, Italy; 9 Division of Obstetrics and Gynecology, Medizinische Hochschule Brandenburg – Immanuelklinik Rüdersdorf, Berlin, Germany; 10 Division of Obstetrics and Gynecology, CHIRMED Department, G. Rodolico Hospital, University of Catania, Catania, Italy; 11 Department of Obstetrics and Gynecology, Biomedical Research Institute of Murcia (IMIB-Arrixaca), Murcia, Spain; 12 Department of Obstetrics and Gynecology, University Hospital Augsburg, Augsburg, Germany; 13 Heilig-Geist Hospital Bensheim, Bensheim, Germany; 14 Department of Obstetrics and Gynecology, Wilhelminen Hospital, Vienna, Austria; 15 Evangelisches Krankenhaus Oberhausen, Wesel, Germany; 16 Department of Medical Education, University of Medical Sciences, Poznan, Poland; 17 Department of Obstetrics and Gynecology, Geneva University Hospitals, Geneva, Switzerland

**Keywords:** Laparoscopic lateral suspension, Sacrocolpopexy, Pelvic organ prolapse, Delphi consensus, Pelvic floor reconstructive surgery

## Abstract

**Introduction and hypothesis:**

Lateral suspension is an abdominal prosthetic surgical procedure used to correct apical prolapse. The procedure involves the placement of a T-shaped mesh on the anterior vaginal wall and on the isthmus or uterine cervix that is suspended laterally and posteriorly to the abdominal wall. Since its description in the late 90s, modifications of the technique have been described. So far, no consensus on the correct indications, safety, advantages, and disadvantages of this emerging procedure has been reached.

**Methods:**

A modified Delphi process was used to build consensus within a group of 21 international surgeons who are experts in the performance of laparoscopic lateral suspension (LLS). The process was held with a first online round, where the experts expressed their level of agreement on 64 statements on indications, technical features, and other aspects of LLS. A subsequent re-discussion of statements where a threshold of agreement was not reached was held in presence.

**Results:**

The Delphi process allowed the identification of several aspects of LLS that represented areas of agreement by the experts. The experts agreed that LLS is a safe and effective technique to correct apical and anterior prolapse. The experts highlighted several key technical aspects of the procedure, including clinical indications and surgical steps.

**Conclusions:**

This Delphi consensus provides valuable guidance and criteria for the use of LLS in the treatment of pelvic organ prolapse, based on expert opinion by large volume surgeons’ experts in the performance of this innovative procedure.

**Supplementary Information:**

The online version contains supplementary material available at 10.1007/s00464-024-10917-5.

Pelvic organs prolapse (POP) affects many women worldwide, causing a significant impact on quality of life [1]. While several transvaginal surgical strategies are available to address this condition, sacral suspension is so far the only transabdominal procedure that has been standardized and thoroughly assessed for safety and efficacy [2]. Based on available evidence, laparoscopic sacrocolpopexy (LSCP) is the gold standard for the correction of advanced apical and multicompartmental POP [3].

LSCP is a challenging procedure associated with rare but potentially severe complications. Sacral dissection can expose to accidental injury of the pre-sacral arteries and veins, of the right hypogastric nerve, ureter, and hypogastric artery [4]. A surgical damage of the left common iliac vein is a life-threatening complication that requires advanced skills to be controlled. An additional post-operative complication is a spondylodiscitis linked to the transfixion of the S1–S2 intervertebral disk during the fixation of the mesh to the overlying longitudinal ligament. In addition, LSCP requires deep dissection skills and placement of stitches on difficult-to-access anatomical areas [5]

In the recent past, new abdominal surgical approaches have been developed to correct advanced apical and multicompartmental POP, including pectopexy [6] and laparoscopic lateral suspension (LLS) [7]. These procedures do not involve sacral dissection and are characterized by shorter learning curves, lower intraoperative risks, and seemingly comparable efficacy in correcting apical prolapse. These techniques turn into different anatomical displacements of the apex and of the vaginal walls, which may imply that these procedures might be useful to tailor the correction of specific types and combination pf pelvic floor defects [8].

LLS corrects pelvic organ prolapse using a T-shaped prosthesis that is attached to the apex and the anterior vaginal wall (in some instances also to the posterior vaginal wall). Then, the two arms of the mesh are pulled laterally and posteriorly towards the abdominal wall. LLS preserves vaginal length and width, allowing women to maintain a normal sexual function. LLS is technically less complex compared to LSCP, as it requires less dissection and less stitching and knot-tying, thus, making it more feasible via a minimally invasive approach [9].

Hence, LLS is gaining popularity as an effective and safe surgical approach to address advanced POP, but the surgical technique has not yet been fully standardized, and its specific indications are not well defined [10]

This article stems from a collaboration of a group of expert pelvic floor surgeons with a broad expertise in the use of LLS, with the aim to build consensus though a Delphi process on the technical aspects, indications, benefits, and pitfalls of this innovative procedure.

## Laparoscopic lateral suspension: surgical technique

Lateral suspension was first described as an open procedure by Kapandji in 1967 as a colpo-isthmo-cystopexy with transverse strips and later modified and repurposed for laparoscopy by Jean-Bernard Dubuisson in 1997 [11].

LLS is typically performed using a T-shaped mesh. The video demonstrates a step-by-step breakdown of the procedure. The mesh is placed and sutured to the anterior vaginal wall and to the uterine isthmus after dissecting the vesicovaginal space. A sub-peritoneal tunnel is created by inserting a laparoscopic instrument laterally and dorsally on the abdomen, about 3–4 cm above and 3 cm dorsally to the anterior–superior iliac spine. The laparoscopic instrument is pushed to reach the peritoneum and then down to the pelvis under the round ligament, to grasp the lateral arm of the mesh, and to pull it out to the skin through the sub-peritoneal tunnel. This procedure performed bilaterally, on right and left sides, such that a hammock-like structure is formed that suspends the uterus and the vagina to the aponeurosis of the oblique muscles, without tension [12].

## Delphi process

The identification and selection of the candidates to participate to the study took place between December 2022 and January 2023. A comprehensive review of the literature was performed so to identify surgeons who had published in peer-reviewed international journals on LLS. Additional candidates were identified through personal referral from experts. The main criteria for being included was a consistent and continued experience in performing LLS in high volumes. After this step, 28 surgeons were invited to participate to the Delphi project. Six experts declined participation and one did not complete all rounds; thus, the final faculty comprised 21 expert surgeons. The group of experts exhibited heterogeneity in terms of gender and country of origin, as indicated in Table 1. Nearly half of the experts were highly experienced and high-volume surgeons, having performed over 100 surgical procedures annually for more than 10 years in the field of pelvic floor reconstruction, encompassing vaginal, open, LPS, and robotic approaches. All faculty members were proficient in LLS. Half of the experts had been performing LLS surgery for 5–10 years, and all surgeons had over 3 years of experience in pelvic floor reconstructive surgery. Three quarters of the panel performed LSCP on a regular basis.

**Table 1:** Descriptive analysis of the participating experts

Category	*N* (%)
*Specialists*	
Gynecologists	21 (100)
*Sex*	
Women	11 (52)
Male	10 (48)
*Countries*	7
Austria	1 (5)
China	1 (5)
Germany	5 (24)
Italy	9 (42)
Poland	1 (5)
Spain	3 (14)
Switzerland	1 (5)
*No of PFRS per year (range)*	
1–50	5 (24)
51–99	7 (33)
≥ 100	9 (43)
*Experience of PF surgeon (years)*	
≤ 4	2 (9.5)
5–9	5 (24)
≥ 10	14 (66.5)
*Surgeons performing LLS*	
Yes	21 (100)
No	0 (0)
*Surgeons performing RLS*	
Yes	8 (38)
No	13 (62)
*Experience of LLS/RLS (years)*	
≤ 4	9 (43)
5–9	10 (47.5)
≥ 10	2 (9.5)
*Surgeons performing LSCP*	
Yes	18 (86)
No	3 (14)
*Surgeons performing RSCP*	
Yes	8 (38)
No	13 (62)

PFRS, pelvic floor reconstructive surgery; PF, pelvic floor; LLS, laparoscopy lateral suspension; RLS, robotic lateral suspension; LSCP, laparoscopic sacrocolpopexy; RSCP, robotic sacrocolpopexy

A scientific committee was formed at the start of the process and consisted of Tommaso Simoncini, Jean Dubuisson, and Friedrich Pauli. The scientific committee was responsible for the generation of 64 statements addressing different aspects of LLS to be submitted to the evaluation of the panel. The statements are reported in Table 2 and are divided into five sub-areas: panel characteristics, indications, surgical technique, particular/complex cases, and post-operative management.

**Table 2:** First round of the Delphi process

Level of agreement (*N* = 21)	
*Indications*	
Lateral suspension requires less surgical expertise than sacrocolpopexy	81%
I choose to perform lateral suspension with TiLoop mesh, over sacrocolpopexy, in obese patients	52%
I choose to perform lateral suspension with TiLoop mesh, over sacrocolpopexy, in patients with previous abdominal surgery	38%
I choose to perform lateral suspension with TiLoop mesh, over sacrocolpopexy, in patients with advanced prolapse	29%
I choose to perform lateral suspension with TiLoop mesh, over other reconstructive techniques, in patients with isolated cystocele (≥ II stage, POP-Q system scale)	10%
I choose to perform lateral suspension with TiLoop mesh, over other reconstructive techniques, in patients with isolated apical defect (≥ II stage, POP-Q system scale)	67%
I choose to perform lateral suspension with TiLoop mesh, over other reconstructive techniques, in patients with combined apical and anterior defect (≥ II stage, POP-Q system scale)	90%
I choose to perform lateral suspension with TiLoop mesh, over other reconstructive techniques, in patients with multicompartmental prolapse (anterior, apical and posterior defect ≥ II stage, POP-Q system scale)	38%
Lateral suspension is the reference procedure to treat a combined anterior and apical defect	90%
Lateral suspension does not correct posterior prolapse	62%
I choose to perform lateral suspension with TiLoop mesh also to treat vaginal vault prolapse	71%
Lateral suspension with TiLoop mesh is not indicated in patients with obstructed defecation symptoms	19%
Lateral suspension performed in patients with anterior and apical defect can facilitate the development of a de novo posterior prolapse	43%
I do not choose to perform lateral suspension with uterus preservation in patients with large uterine fibroids or adenomyosis	71%
Lateral suspension is as effective as sacral suspension in restoring advanced apical prolapse	76%
In case of apical and anterior defect I choose to perform lateral suspension over sacrocolpopexy because it is a procedure that avoids the dangers and technical challenges of sacral mesh fixation	62%
I choose to perform lateral suspension with TiLoop mesh over sacrocolpopexy in the belief that placement of a mesh reinforcement of the vescicovaginal space and a lateral traction of the vaginal axis allows a better anatomic correction of the apical–anterior prolapse	81%
I choose to perform lateral suspension as a salvage strategy to manage apical prolapse relapse in patients that were previously treated with sacrocolpopexy	71%
I choose to perform lateral suspension in case of patients who want to preserve the uterus	90%
Lateral suspension allows a more anatomically effective restoration of the position of the apex compared to sacral suspension	62%
*Surgical technique*	
The use of a uterine manipulator during the procedure is required	52%
Vaginal retractor is helpful to expose and dissect the vescicovaginal septum	90%
The vesicovaginal septum dissection has ideally to be performed down to the level of the bladder trigone	86%
I perform the cutaneous incision for the retroperitoneal surgical step	62%
I cut the lateral arms at skin level without tensioning the mesh	52%
After tunneling the side arms of the mesh, I do not always attach them to the abdominal muscle fascia	76%
After tunneling the side arms of the mesh, I attach them to the abdominal muscle fascia only in selected cases (obesity for example)	38%
After tunneling the side arms of the mesh, I consider sufficient to adjust the tension manually	86%
How many stitches or tackers do you usually use to attach the mesh to the anterior vaginal wall?	52%
A key element for the efficacy of the procedure stays in a solid fixation of the mesh to the anterior vaginal wall and apex	95%
When I perform lateral suspension, I often/ always combine a concomitant transvaginal posterior compartment correction	19%
When I perform lateral suspension, I do not often/always combine a concomitant trans-rectal posterior compartment correction	81%
When I perform lateral suspension, I do not insert a transabdominal mesh at the level of the recto-vaginal septum	76%
When I perform lateral suspension, I use a pre-shaped mesh	81%
When I perform lateral suspension, I combine a supracervical hysterectomy only in selected cases	67%
When I perform lateral suspension, I do not always combine a supracervical hysterectomy in every patient	90%
When I perform lateral suspension and a supracervical hysterectomy, I prefer to use titanized mesh implant TiLOOP^®^ LLS Dubuisson with anterior flap	57%
When I perform a supracervical hysterectomy at the same time of lateral suspension, I prefer to use titanized mesh implant TiLOOP^®^ LLS H Dubuisson with anterior and posterior flap	62%
When I perform lateral suspension for the treatment of vaginal vault prolapse, I prefer to use titanized mesh implant TiLOOP^®^ LLS H Dubuisson with anterior and posterior flap	71%
*Open questions:*	
1. I usually attach the mesh to the anterior vaginal wall with (more than one option): Delayed absorbable material (67%) Tackers (38%) Monofilament suture (29%) Non absorbable material (14%) Fast absorbable material (10%) Multifilament suture (10%) Glue (0%)	
2. I usually attach the mesh to the isthmus with: Nonabsorbable material (90.5%) Multifilament suture (24%) Monofilament suture (19%) Delayed absorbable material (14%) Tackers (14%) Fast absorbable material (0%) Glue (0%)	
*Particular cases and surgical skills*	
In patients with anterior defect relapse after lateral suspension, a distal cystocele can be corrected transvaginally with anterior colporrhaphy	86%
In case of relapse of apical prolapse after lateral suspension, I try a laparoscopic re-suspension of the lateral arms of the same mesh	24%
In case of relapse of apical prolapse after lateral suspension I consider treating the prolapse by transvaginal route	19%
In case of relapse of apical prolapse I never observed a detachment of the mesh from the vagina or from the cervix	57%
In case of relapse of apical prolapse after lateral suspension, I decide not to remove the previously placed mesh but to refix and retension it	52%
In case of relapse of apical prolapse after lateral suspension I decide to remove previously placed mesh and to perform a sacral suspension	29%
The incidence of mesh-related complications after lateral suspension is comparable to that of sacral suspension	29%
*Post-operative management*	
Lateral suspension is associated with risk of de novo stress urinary incontinence	19%
Lateral suspension for pelvic organ prolapse allows earlier discharge of patients than sacrocolpopexy	24%
Lateral suspension for pelvic organ prolapse allows faster urinary recovery than sacrocolpopexy	19%
Lateral suspension for pelvic organ prolapse allows a lower rate of urinary hesitancy than sacrocolpopexy	19%
Lateral suspension for pelvic organ prolapse allows a lower incidence of urinary urgency than sacrocolpopexy	5%
Lateral suspension is associated with a lower risk of recurrent cystocele than sacrocolpopexy	48%

The first round was performed online using the Google Forms application. Each statement was to be scored by the panel’s members based on a 0–10 scale, to indicate the level of agreement or disagreement. A score of 0 indicated total disagreement, while a score of 10 indicated total agreement. The results were analyzed, identifying statements where 70% or more of the group either strongly disagreed (0–3) or strongly agreed (7–10). This resulted in 16 agreements (score 7–10), 5 disagreements (score 0–3), and 33 items where a consensus was not reached (score 4–6), as indicated in Table 2.

A second Delphi round took place in person on May 7th, 2023, in Pisa, Italy. During this round, statements where a trend towards consensus had been reached (60–70% agreement) were reevaluated and resubmitted. As the discussion progressed, new statements emerged, leading to the production of 31 new items that were the panel reached consensus, as indicated in Table 3.

**Table 3:** Second round of the Delphi process

Level of agreement (*N* = 21)	
*Indications*	
I don’t consider morbid obesity a contraindication to LLS	100%
LLS is not the first-line indication for the treatment for primary isolated cystocele	100%
LLS can be considered for the treatment of recurrent isolated cystocele	100%
LLS can be considered in patients with high risk of recurrence (e.g., obesity, diabetes, COPD, ODS, occupational risks, connective tissue disorders…) of recurrence in the presence of primary cystocele	100%
LLS is an option for the treatment of isolated primary hysterocele	100%
I prefer LLS for hysteropexy over ASC-H hysteropexy, because of technical challenges with sacral hysteropexy	100%
LS as a single procedure may not be the best option to treat multicompartmental prolapse	95%
In the presence of vaginal vault prolapse a posterior defect can be treated using the LLS double flap mesh	100%
Treatment of posterior prolapse with LLS is not standardized	100%
Lateral suspension does not seem to be associated with more de novo high posterior compartment defects compared to other abdominal suspending techniques	100%
A significant advantage of LLS over ASC is the avoidance of surgical risks related to the dissection of the promontorium	100%
Vaginal axis and location of the apex after LLS are more similar to normal anatomy compared to ASC	100%
*Surgical technique*	
The use of a vaginal retractor is optimal to perform LLS	100%
Deep dissection of the vescicovaginal septum to the level of the bladder trigone is optimal to correct anterior prolapse	100%
The cutaneous incision for the retroperitoneal tunneling of the mesh should be performed 4 cm above the ASIS and 3 cm laterally in the absence of pneumoperitoneum	100%
The lateral arms of the mesh should be pulled one against the other after removal of any vaginal retractor/manipulator so to achieve a central position of the apex. The lateral arms should be cut at the level of the skin. This provides a suspension that is generally appropriate in most patients. Vaginal inspection is useful to tailor the traction	100%
Fixation of the lateral arms of the mesh to the abdominal muscles’ fascia is not required and should be avoided since it can provide pain	100%
The anterior flap of the mesh should be fixed to the anterior vaginal wall in flat position with a minimum number of 4 fixation points	100%
Mesh fixation to the vaginal wall should be performed with delayed resorbable material	100%
When LLS is perfomed to correct apical and anterior prolapse in the absence of posterior prolapse a transvaginal or transabdominal prophylactic posterior correction is not required	100%
LLS should be performed with pre-shaped titanized mesh specifically designed for this procedure since all available efficacy and safety data refer to these materials	100%
Supracervical hysterectomy is not required for LLS	100%
In the case a supracervical hysterectomy is required it is not necessary to use a double flap mesh to perform LLS	100%
*Particular cases and surgical skills*	
In case of apical failure after LLS the first-line approach for optimal management should be abdominal	100%
In case of apical failure after LLS the first-line approach for optimal management should be abdominal	100%
In case of management of apical relapse after LLS with transvaginal surgery it is not necessary to remove the previous mesh	100%
Mesh-related complications after LLS are rare and similar to those after ASC	100%
*Post-operative management*	
Risk of de novo SUI is not specifically increased with lateral suspension	100%
LLS does not shorten length of hospitalization compared to L/RSC	100%
Recovery of urinary function is similar after LLS or SC	100%
Based on clinical experience The experts concur that LLS seems more efficient in anatomically restoring cystocele compared to ASC	100%

## Data statements and informed consent

The data presented in this article consist of voting statements obtained from a Delphi study. Because no patients were enrolled in the study, local ethics committee approval was not required. Informed consent was considered not applicable in this context. However, ethical considerations were an integral part of the study, with emphasis on maintaining process integrity, transparency, and accountability in reporting. The authors, as participants, thoroughly reviewed and accepted the article for submission.

## Statistical analysis

Categorical variables are expressed as absolute values and frequency, *n* (%). All analyses were performed with IBM SPSS Statistics v.27 technology (SPSS Inc., Chicago, IL).

## Results

### Indications of LLS

The first set of Delphi statements explored the panel’s opinion on the appropriate surgical indications for LLS. The group agreed that LLS requires less surgical expertise compared to LSCP. Indeed, the experts preferred LLS for hysteropexy over LSCP mostly because of the avoidance of surgical risks associated with promontory dissection.

The experts agreed that LLS is as effective as LSCP in correcting isolated advanced apical prolapse and for the treatment of vaginal vault prolapse. In the presence of vaginal vault prolapse, the experts agreed that LLS can be more effective if performed with a double flap mesh fixed to both the anterior and posterior vaginal wall. Furthermore, while LLS is particularly suitable in cases where patients wish to preserve the uterus, the experts agreed that effective apical suspension with LLS may benefit from supracervical hysterectomy in the presence of a bulky/heavy uterus.

According to the expert panel, LLS can be used as a salvage strategy for managing apical prolapse relapse in patients who have previously undergone LSCP.

From an anatomical standpoint, the panel agreed that the vaginal axis and the position of the apex after LLS is closer to normal anatomy than after LSCP since there is less posterior displacement of the apex. To this extent, experts agreed that they prefer LLS to LSCP to treat combined apical/anterior prolapse as they experience better correction of anterior defects with this technique compared to LSCP. Although they considered LLS not to be the primary option for isolated cystocele, yet they considered it a possible approach for recurrent isolated cystocele.

The panel reached a consensus based on their experience that women with minimal or absent posterior defects treated with LLS do not appear to have a higher incidence of de novo posterior prolapse. However, the panel agreed that LLS may not be suitable for the treatment of multicompartmental prolapse due to limited data on its effectiveness in correcting advanced posterior defects.

Based on experts’ opinion, obesity should not be considered a contraindication for LLS.

### Surgical technique

In the second section of the Delphi, the experts' opinions on the technical performance of LLS were examined.

According to the panel, LLS should be performed using a pre-shaped titanized mesh specifically designed for this procedure, as all available data on efficacy and safety are based on this material. The anterior flap of the mesh should be fixed to the anterior vaginal wall in a flat position, utilizing a minimum of 4 fixation points. The use of a vaginal retractor was deemed optimal for performing LLS as it aids in the dissection and exposure of the vesicovaginal septum and of the anterior vaginal wall, ideally to the level of the bladder trigone.

Three quarters of the experts suture the mesh to the anterior vaginal wall using delayed resorbable material (mostly monofilament) or with resorbable tackers, while non-resorbable multifilament material is preferred for suturing the mesh to the uterine cervix and isthmus by most experts.

The panel agreed that the site of the skin incisions for the retroperitoneal tunneling of the mesh is critically important and that it should be located 4 cm above the anterior–superior iliac spine and 3 cm laterally (dorsally) in the absence of pneumoperitoneum. During the retroperitoneal introduction of the grasper and while retracting the mesh arm care should be taken to identify and avoid the gonadal vessels and the external iliac vessels; however, a recent systematic review of the published papers on lateral suspension did not highlight severe complications associated with this surgery or more specifically with this step [10, 13]. The lateral arms of the mesh should be pulled simultaneously after the removal of any vaginal retractor or manipulator to achieve a central position of the apex, and they should be cut at the level of the skin. This provides a suspension that is generally suitable for most patients. The panel agreed that vaginal inspection can be useful to tailor the traction and fixation of the lateral arms of the mesh. The experts concurred that fixation of the prosthesis to the abdominal muscle fascia is not required and should be avoided, as it can cause pain.

### Particular cases and surgical skills

In the third section of statements, the experts' opinions on specific surgical cases and surgical skills related to LLS were examined. According to the experts, if an anterior cystocele occurs after LLS, it can be effectively treated transvaginally with anterior colporrhaphy. However, in the case of apical relapse after LLS, the experts recommended an abdominal approach as the first-line treatment. During such re-do abdominal surgeries, the experts agreed that removal the previous mesh material is not mandatory in the absence of mesh-related complications.

### Post-operative management

In the fourth and final section of statements, the experts' opinions on the post-operative management of LLS were tested. According to the experts, LLS does not result in a shorter hospitalization period compared to LSCP. The risk of de novo stress urinary incontinence (SUI) is not increased with LLS, and the recovery of urinary function is similar after LLS or LSCP.

## Future perspectives

General feelings of the experts were that additional prospective trials focusing on various LLS procedures based on real-life indications should be performed. These trials should aim to evaluate key performance indicators (KPIs) to assess the effectiveness and outcomes of the procedures both with laparoscopic and robotic approaches. The suggested KPIs include POP-Q (Pelvic Organ Prolapse Quantification) measurements at 6 months post-surgery, overall outcomes, complication rates, feasibility, safety, quality of life, sexual function, urinary and bowel function, failure rates, need for re-intervention, timing of complications, and mesh-related complications [14].

By conducting these trials and evaluating these KPIs, the panel thought that valuable data on the performance and impact of LLS could be gathered, hence, obtaining insights into the benefits, risks, and long-term outcomes associated with this procedure and allowing for evidence-based decision-making and further refinement of surgical technique. In addition, the proficiency of surgeons in performing LLS should also be evaluated in these studies to ensure optimal patient outcomes [15].

## Discussion

This consensus highlights several important points on the technique and the indications of LLS as a surgical procedure for the treatment of pelvic organ prolapse [16].

LLS represents a significant advancement in the field of abdominal POP surgery, offering an alternative to sacral suspension. As with any surgical technique, ongoing research is needed to refine and improve the procedure, to enhance its outcomes, and to delineate better its indications [17].

LSCP is the gold standard for the correction of apical prolapse. However, according to the experts' opinion and to a recent randomized non-inferiority trial, LLS is equally effective to LSCP in correcting apical and anterior prolapse [18].

Thus, LLS is a valuable new tool with growing literature evidence that can be used to treat advanced pelvic organ prolapse. This is particularly important, as it expands the surgical armamentarium and may provide the chance to better tailor the type of suspension (i.e., sacral vs. lateral) based on the type of prolapse [19]. Indeed, based on the experience of the participants to the Delphi process, lateral suspension is more effective in correcting an advanced anterior prolapse compared to sacral suspension. Thus, if this is confirmed with future trials, LLS may not represent a simple alternative to LSCP, but rather a procedure aimed at the preferential treatment of apical/anterior defects, while LSCP may be more appropriate for the management of apical/posterior or three-compartmental prolapses [20].

In addition, mastering LLS allows the management of those rare cases where the sacral promontorium is not accessible or of recurrences or complications of a previous sacrocolpopexy, where a re-do attachment of a mesh to the promontorium is often not feasible or contraindicated (e.g., in the presence of mesh complications).

As a further advantage, the surgical skills required for the performance of LLS are simpler than those needed to perform LSCP, as confirmed by the panel of experts. This results in a quicker acquisition of expertise and in a potential broader diffusion of the procedure, hence in a potential advantage as a valuable and effective surgery might be made available to a broader population [21].

Yet, the experts highlighted through their agreement and discussion how LLS is not a simple procedure and that it requires specific technical attentions. Specifically, a suitable mesh needs to be used and the standard is the titanized, T-shaped polypropylene mesh that has been used to generate the evidence on this procedure [22]. In addition, specific recommendations on the number and type of sutures to secure the mesh were provided by the experts. Finally, the experts deemed critical that the suspension needs to be directed laterally and posteriorly by identifying a specific location to introduce the laparoscopic instrument used to pull the mesh [23].

According to the experts' opinion, in the future, there may be space to explore further a wider range of mesh options that could be used to further tailor lateral suspension, including different lengths and widths of mesh arms, which would allow for greater adaptability to various types of prolapse, and possibly to correct multicompartmental defects [24].

The experts called for randomized controlled trials to compare different surgical approaches for the treatment of POP to consolidate the advantages and disadvantages of each procedure and the appropriate surgical indications [25].

## Conclusions

By providing shared expert opinion and recommendations based on common experience, the committee aimed to provide other pelvic surgeons with valuable information on the role, the safety, and the strengths and pitfalls of LLS compared to the current gold standard, LSCP [26, 27]. Based on this consensus, LLS emerges as a suitable, safe, and effective procedure to treat advanced apical prolapse that requires attention and further clinical development to fully understand its surgical place in the treatment of pelvic floor defects.

### Supplementary Information

Below is the link to the electronic supplementary material.

Supplementary file1 (MP4 173359 KB)

## Abbreviations

PFRS: Pelvic floor reconstructive surgery
PF: Pelvic floor
POP: Pelvic organ prolapse
LLS: Laparoscopic lateral suspension
LSCP: Laparoscopic sacrocolpopexy
